# The Influence of Service Temperature and Thickness on the Tensile Properties of Thin T2 Copper Sheets

**DOI:** 10.3390/ma15072341

**Published:** 2022-03-22

**Authors:** Yebao Ge, Ruibin Gou, Min Yu, Chunyu Zhang, Nian Wang, Hao Xu

**Affiliations:** 1 College of Mechanical Engineering, Anhui Science and Technology University, Fengyang 233100, China; geyebao8080@163.com (Y.G.); gourb@ahstu.edu.cn (R.G.); chunyu_zh@163.com (C.Z.); xuhao0889@163.com (H.X.); 2 School of Mechanical Engineering, Anhui Polytechnic University, Wuhu 241000, China; 3 College of Architecture, Anhui Science and Technology University, Bengbu 233000, China; 4 Bengbu Special Equipment Supervision and Inspection Center, Bengbu 233000, China; wangjun74011@163.com

**Keywords:** thin T2 copper sheets, tensile properties, service temperature, predict model

## Abstract

Thin T2 copper sheets with nine different thicknesses were employed in uniaxial tensile tests to investigate the influence of service temperature and thickness on their tensile properties. A total of 33 groups of tensile samples were separately tested at 20 °C, 100 °C, 150 °C, 200 °C, and 250 °C to obtain their elongation and their tensile and yield strengths. The change laws of the tensile properties of the investigated T2 copper were analyzed using different fitting functions. The main results show that both sheet thickness and temperature have an important influence on the tensile properties of T2 copper. As the sheet thickness increased, the tensile and yield strengths of the tested materials first increased rapidly, then decreased sharply, and finally stabilized. As the temperature increased, the tensile strength increased linearly while the yield strength decreased linearly. The relationships between the elongation and the sheet thickness and temperature were exponential and polynomial functions, respectively. *T*–*t*–*Rm*, *T*–*t*–*Rel*, and *T*–*t*–*δ* empirical formulas were proposed and established to predict the tensile properties of the investigated T2 copper sheet, and the predictive models exhibited solid accuracy.

## 1. Introduction

With the rapid development and the improvement of the accuracy requirements in many science fields, micromechanical systems (MEMS) have developed rapidly, and the demand for miniaturization of materials is also increasing year by year [1,2,3]. Because of the excellent electrical conductivity and ductility of copper and its alloys, they are widely used in micro-parts of precision instruments [4,5,6]. Unfortunately, when the sheet thickness reaches the accuracy of millimeters and below, the mechanical properties often show a size effect which differs from that of conventional materials, and the phenomenon becomes more obvious with decreasing sheet thickness [7,8]. Moreover, as the sheet thickness decreases, the influence of the service temperature on the performance of the material might become more pronounced and cannot be ignored.

In recent years, many workers have identified the phenomenon that, when the thickness is small, the mechanical properties of copper and its alloys are contrary to those of conventional materials. It was found that, when the sheet thickness is above the millimeter scale, the relevant mechanical properties of sheets of materials such as aluminum and steel deteriorate as the thickness of the sample decreases [9,10,11]. When the sheet size is on the micrometer scale, researchers found that different materials exhibit different mechanical properties compared to conventional materials. Wu et al. [12] studied the mechanical behavior of 304 stainless steel and found that the yield strength first increased and then decreased, while the tensile strength and elongation showed opposite properties; the finite element model of rigid die bulging was established. Wan et al. [13] applied a self-developed micro asymmetric mill to produce a CP–copper ultrathin strip, revealing that the tensile strength and hardness first increased and then decreased with the decrease of thickness. Zhang et al. [14] carried out tensile test on a 0.1–1.0 mm copper sheet. The test results showed that the tensile strength, the maximum uniform strain, and the fracture strain decreased with the decrease in sheet thickness, while the yield strength exhibited the opposite trend. Lee [15] and Yu et al. [16] found that the yield strength increased with the decrease in thickness in the tensile test of 0.1–10 µm metal film. Li et al. [17,18] carried out microscale bending experiments on pure aluminum foil and pure copper with different thicknesses. The experimental results showed that the spring-back angle increased with the decrease in thickness, while the flow stress increased with the decrease in foil thickness. A change in temperature also has a huge impact on the mechanical properties of the material. He et al. [19] tested the effect of heat treatment on 304 stainless steel. The test results indicated that the strength and elongation were decreased after heat treatment. Meng et al. [20] heat-treated pure copper of different thicknesses and found that the mechanical properties of thinner samples of pure copper decreased with heat treatment, while the opposite was seen for thicker samples of pure copper. Kori et al. [21] conducted room-temperature and high-temperature (100 °C, 200 °C, 300 °C) tensile tests on 304 stainless steel samples and found that the yield strength and elongation decreased initially and then increased within the test range, while the tensile strength value decreased. Zhang et al. [22] performed room- and cryogenic-temperature tensile tests on pure copper samples after heat treatment, finding that the strength and ductility obtained at −196 °C were higher than for tensile samples deformed at 22 °C. At present, there have been many achievements in the research on the influence of thickness and heat treatment on the mechanical properties of thin sheets, and the influence of service temperature on material properties has gradually been elucidated. However, the related research on the joint effect of sheet thickness and service temperature on the mechanical properties of sheets is still rare.

In the present study, a thin T2 copper sheet was employed to investigate the effect of thickness and service temperature on its tensile properties. To investigate the change law of tensile properties referring to tensile strength, yield strength, and elongation, uniaxial static tensile tests were performed on T2 copper sheets of various thicknesses from 0.08 mm to 1.0 mm at different test temperatures. Moreover, variables of sheet thickness and test temperature were defined so as to establish their relationships with tensile properties.

## 2. Experiments

### 2.1. Materials and Samples

In order to systematically study the effect of sheet thickness on the tensile properties of the tested materials, thin T2 copper sheets of nine different thicknesses were employed in the present work, and their main chemical composition is shown in Table 1. The thicknesses of the employed T2 copper sheets were 0.08 mm, 0.15 mm, 0.20 mm, 0.25 mm, 0.30 mm, 0.35 mm, 0.60 mm, 0.80 mm, and 1.0 mm.

**Figure 1:**
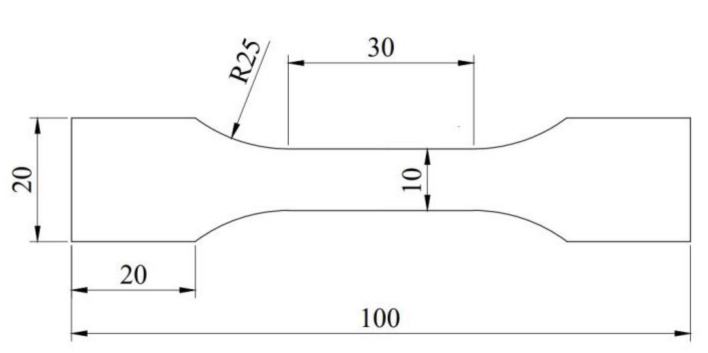
The dimensions of the tensile sample (mm).

The dimensions of the standard tensile specimen are shown in Figure 1.

### 2.2. Uniaxial Static Load Tensile Test

Using a 100 kN electronic universal testing machine and an environmental box (test temperature range: −70 to +350 °C, temperature deviation: ≤±2.0 °C), uniaxial static load tensile tests were performed on the tested T2 copper sheets with different thicknesses under different temperature conditions. The tensile speed was 1 mm/min, and the test temperatures were 20 °C (normal temperature), 150 °C, and 250 °C. Additionally, three sets of samples and two test temperatures were added: 0.20 mm thick, 0.60 mm thick, and 1.0 mm thick copper sheets at 100 °C and 200 °C. Before testing, the specimen was heated to the service temperature with a furnace and then held for 10 min. All samples were divided into 33 groups according to sheet thickness and test temperature, and five specimens were prepared for each group, as shown in Table 2.

## 3. Results

In order to ensure the reliability of the test results, at least three samples were tested for each group. The yield strengths, tensile strengths, and elongations of each group samples were obtained and investigated.

### 3.1. Typical Stress–Strain Curve of the Investigated T2 Copper Sheets

**Figure 2:**
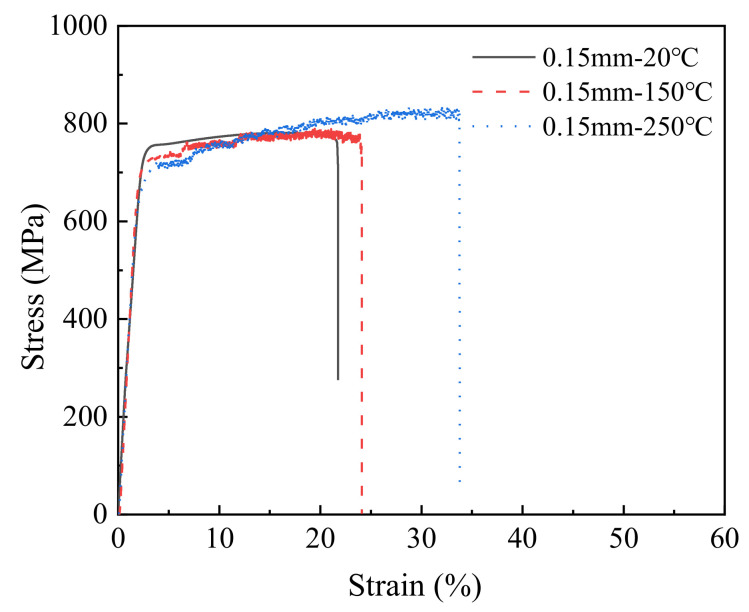
Stress–strain curves of the 0.15 mm thick copper sheet under different temperatures.

Because the results of various T2 copper sheets were basically the same, the stress–strain curves of the 0.15 mm thick T2 copper sheet are given as a representative example, as shown in Figure 2.

### 3.2. The Tensile Properties of the Investigated T2 Copper Sheets

**Figure 3:**
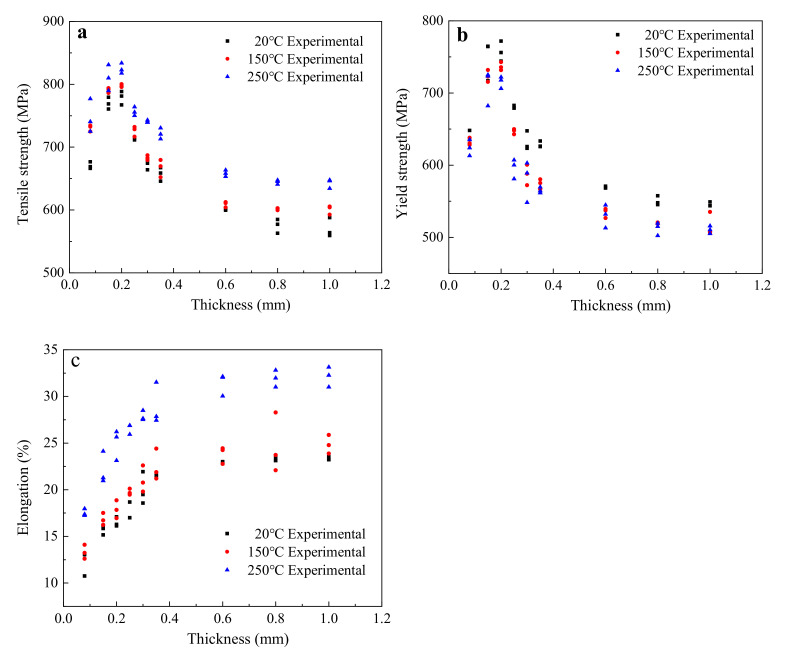
Results of the tensile properties of the nine investigated T2 copper sheets: (**a**) tensile strength; (**b**) yield strength; (**c**) elongation.

The tensile strength, yield strength, and elongation of each sample were obtained and investigated, as shown in Figure 3.

Figure 3 indicates that the change laws of tensile properties were almost the same under different temperatures, with an obvious influence of sheet thickness on the tensile properties of T2 copper independent of the test temperature. The effect of sheet thickness on tensile strength and yield strength was almost the same. When sheet thickness was less than 0.20 mm, the tensile strength and yield strength of the T2 copper increased rapidly with the increase in the sheet thickness. However, when sheet thickness was greater than 0.20 mm, the tensile strength and yield strength of the T2 copper firstly decreased sharply and then stabilized with the increase in sheet thickness. For sheet thickness in the range of 0.08 mm to 1.0 mm, the value of elongation gradually increased at first and then stabilized.

## 4. Analysis and Discussion

The curve fitting method was implemented to reveal the influence of both the sheet thickness and the service temperature on the tensile properties of the investigated T2 copper sheets.

### 4.1. The Sheet Thickness Effect on the Tensile Mechanical Properties of T2 Copper Sheets

#### 4.1.1. The Influence of the Sheet Thickness on Tensile Strength

**Figure 4:**
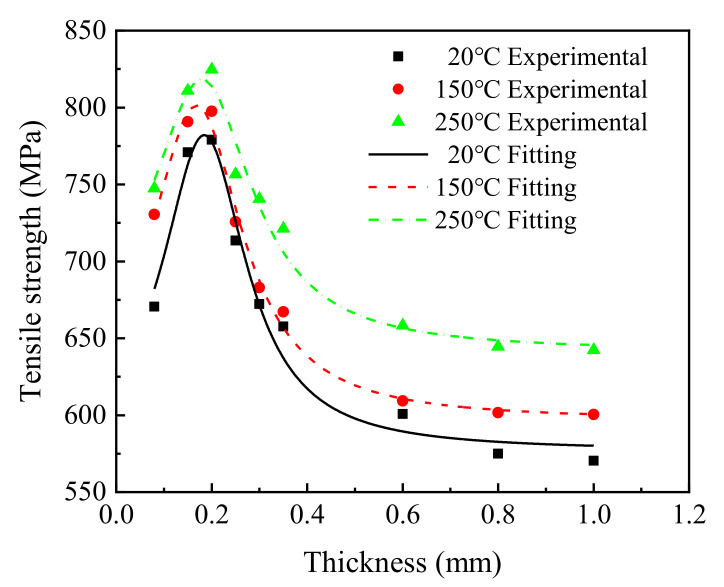
The change law of the tensile strength of T2 copper sheet under different temperatures.

To reveal the influence of sheet thickness on the tensile strength of T2 copper sheets, the average tensile strengths of the 27 groups at three temperatures were analyzed using the Lorentz function fitting method (Equation (1)), and the experimental and fitting results are shown in Figure 4 and Table 3.

Figure 4 shows that, when the sheet thickness was less than 0.20 mm, the tensile strength of T2 copper increased from 670 MPa to 779 MPa at 20 °C, from 773 MPa to 797 MPa at 150 °C, and from 747 MPa to 825 MPa at 250 °C. However, when the sheet thickness was greater than 0.20 mm, the tensile strength of T2 copper decreased from 779 MPa to 570 MPa at 20 °C, from 797 MPa to 600 MPa at 150 °C, and from 825 MPa to 642 MPa at 250 °C.

The above results indicate that the T2 copper sheet showed a phenomenon of increasing strength with decreasing thickness for thickness values greater than 0.20 mm, but exhibited the opposite phenomenon for sheet thickness values less than 0.20 mm, similar to the results in [13], with slightly deviation due to the difference in trace elements of the material composition.

The relationship between tensile strength and thickness of the T2 copper sheet was fitted using the Lorentzian function.

(1) $$y=y_{0}+\frac{2A}{\pi }\left(\frac{\omega }{4{\left(t-t_{0}\right)}^{2}+\omega^{2}}\right),$$

where *y* is the function value, *y*_0_ is the offset, *A* is the area, *t* is the sheet thickness, *t*_0_ is the center, and *ω* is the half-width.

According to Table 3, parameters *A*, *t*_0_, and ω can be regarded as temperature-independent constants because they differed slightly at different temperatures. However, parameter *y*_0_ can be regarded as a temperature-dependent variable because its fitted value varied significantly, and it could be determined using the experimental tensile strength of the investigated material with maximum thickness at the test temperature.

Therefore, substituting the fitted average values of parameters *A*, *t*_0_, and *ω* into Equation (1), i.e., *A* = 71.968, *t*_0_ = 0.179, and *ω* = 0.231, a relationship between sheet thickness *t* and the material tensile strength *R_m_* could be obtained as follows:

(2) $$R_{m}=R_{m0}+\left(\frac{10.584}{4{\left(t-0.179\right)}^{2}+0.053}\right),$$

where *R_m_* is the tensile strength of the investigated T2 copper sheet, *R*_*m*0_ is a temperature-dependent variable approximately determined using the experimental tensile strength of the thickest T2 copper sheet at the test temperature, and *t* is the sheet thickness.

#### 4.1.2. The Influence of the Sheet Thickness on Yield Strength

**Figure 5:**
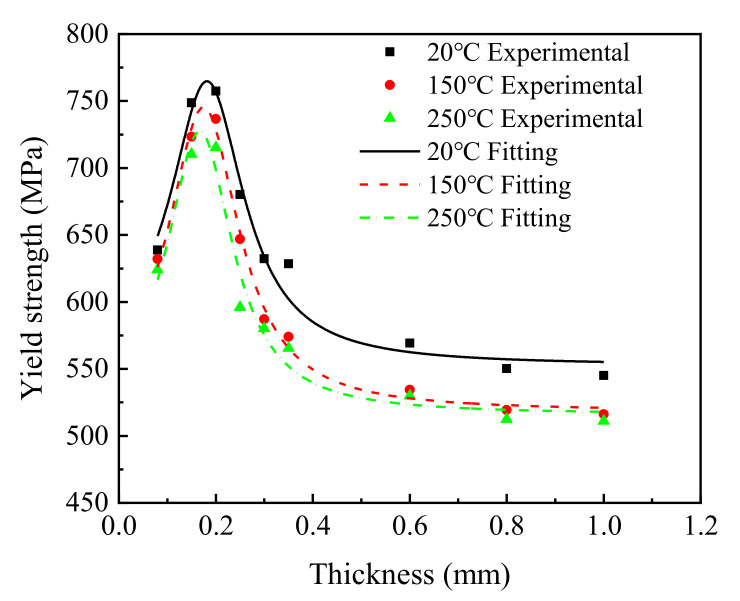
The change law of the yield strength of T2 copper sheet under different temperatures.

To reveal the influence of sheet thickness on the yield strength of the T2 copper sheet, the average yield strengths of all 27 samples in three temperature groups were fitted using the Lorentz function, and the fitting results are shown in Figure 5 and Table 4.

As shown in Figure 5, when the sheet thickness was less than 0.20 mm, the yield strength of the T2 copper increased rapidly from 639 MPa to 757 MPa at 20 °C, from 632 MPa to 736 MPa at 150 °C, and from 624 MPa to 715 MPa at 250 °C. When sheet thickness was greater than 0.20 mm, the yield strength decreased from 757 MPa to 545 MPa at 20 °C, from 736 MPa to 516 MPa at 150 °C, and from 715 MPa to 511 MPa at 250 °C, respectively. This trend of yield strength first increasing and then decreasing with increasing thickness is consistent with previous results using 304 stainless steel [12].

According to Table 4, similar to tensile strength, parameters *A*, *t*_0_, and ω could be regarded as temperature-independent constants, whereas parameter *y*_0_ could be regarded as a temperature-dependent variable. The value of the parameter *y*_0_ could be determined using the experimental yield strength of the investigated material with maximum thickness at the test temperature.

Therefore, substituting the fitted average values of parameters *A*, *t*_0_, and ω into Equation (1), i.e., *A* = 61.811, *t*_0_ = 0.178, and *ω* = 0.180, a relationship between sheet thickness *t* and the material yield strength *Rel* could be obtained as follows:

(3) $$Rel=Rel_{0}+\left(\frac{7.083}{4{\left(t-0.178\right)}^{2}+0.032}\right),$$

where *Rel* and *t* are the yield strength and thickness of the investigated T2 copper sheet, and *Rel*_0_ is a temperature-dependent variable approximately determined using the experimental yield strength of the thickest T2 copper sheet at the test temperature.

#### 4.1.3. The Influence of the Sheet Thickness on Elongation

**Figure 6:**
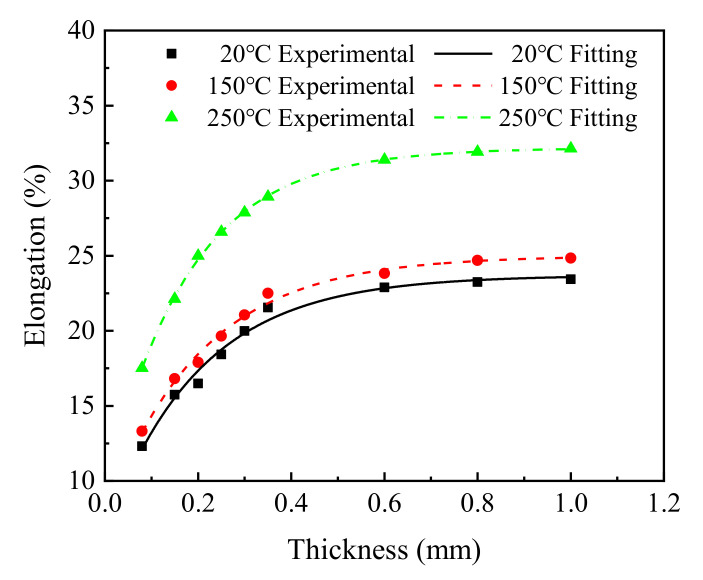
The change law of elongation of T2 copper sheet at different temperatures.

As shown in Figure 3c, the average elongation of each group of samples was calculated. According to the calculated results, the exponential function given in Equation (4) was used to fit the experimental results, as shown in Figure 6 and Table 5.

Figure 6 shows that the elongation gradually increased and then stabilized, which is in good agreement with the results reported by Zhou et al. [23]. With increasing sheet thickness, the elongation of the T2 copper increased from 12.31% to 23.43% at 20 °C, from 13.31% to 24.85% at 150 °C, and from 17.52% to 32.13% at 250 °C.

The relationship between the elongation of T2 copper sheet and the thickness of the sheet was fitted by an exponential function.

(4) $$y=y_{0}-a\;\cdot \;b^{t},$$

where *y* is the elongation of T2 copper, *y*0 is the offset of the elongation, *a* and *b* are two material parameters, and t is the sheet thickness.

As shown in Table 5, parameters *a* and *b* could be regarded as temperature-independent constants, whereas parameter *y*_0_ could be regarded as a temperature-dependent variable. The value of parameter *y*_0_ could be approximately determined using the experimental elongation of the investigated material with maximum thickness at the test temperature.

Therefore, substituting the fitted average values of parameters *a* and *b* into Equation (4), i.e., *a* = 19.585 and *b* = 0.005, a relationship between sheet thickness *t* and the elongation *δ* could be obtained as follows:

(5) $$\delta =\delta_{0}-19.585\times 0.005^{t},$$

where *δ* and *t* are the elongation and thickness of the investigated T2 copper sheet, and *δ*_0_ is a temperature-dependent variable approximately equal to the average experimental elongation of the thickest T2 copper sheet at the test temperature.

### 4.2. The Influence of Service Temperature on Tensile Properties of T2 Copper Sheet

To comprehensively study the effect of service temperature on the T2 copper sheet tensile properties, another six groups of samples and two test temperatures were added introduced according to Table 2: 0.20 mm thick, 0.60 mm thick, and 1.0 mm thick copper sheets at 100 °C and 200 °C.

#### 4.2.1. The Influence of Service Temperature on Tensile Strength

**Figure 7:**
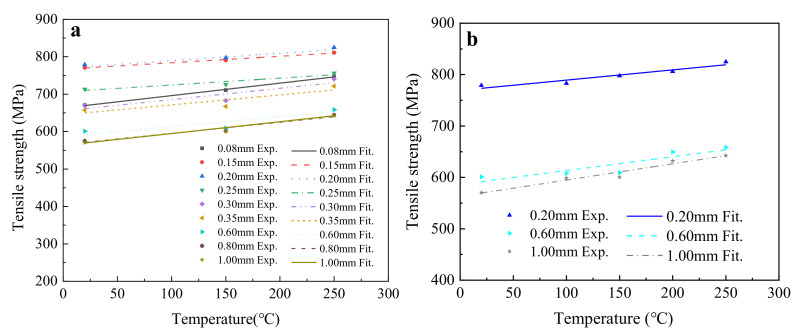
The influence of temperature on the tensile strength of T2 copper sheet: (**a**) fitting results for three temperatures; (**b**) fitting results for five temperatures.

To reveal the influence of service temperature on the tensile strength of T2 copper sheets, the average tensile strengths were analyzed using a linear function fitting method (Equation (6)), and the experimental and fitting results are shown in Figure 7 and Table 6.

(6) $$y=a_{1}+b_{1}\times T,$$

where *y* is the value of the fitted parameter, *a*_1_ is the intercept, *b*_1_ is the slope, and *T* is the temperature.

Figure 7 indicates that the change laws of tensile strength were almost the same for T2 copper sheets of different thickness, revealing an obvious increasing linear relationship with temperature. Table 6 indicates that the fitting results of five temperatures were basically consistent with those of three temperatures, with a maximum fitting deviation of 10% for the 0.6 mm thick T2 copper sheet. Thus, the fitting results of three temperatures were reliable and accurate.

According to the *Rm*_0_ in Equation (2), the tensile strength of the 1.0 mm thick T2 copper sheet could be obtained by substituting *y* = *Rm*_0_, *a*_1_ = 563.204, and *b*_1_ = 0.316 into Equation (6), as given in Equation (7).

(7) $$R_{m\;t\;=\;1.0}=R_{m0}=563.204+0.316T,$$

where *Rm*_0_ is the tensile strength of the T2 copper sheet, and *T* is the temperature.

Substituting Equation (7) into Equation (2), a service temperature–thickness–tensile strength (*T*–*t*–*Rm*) empirical formula of T2 copper sheet could be established, as shown in Equation (8).

(8) $$R_{m}=563.204+0.316T+\left(\frac{10.584}{4{\left(t-0.179\right)}^{2}+0.053}\right),$$

where *Rm*, *T*, and *t* are the tensile strength of the T2 copper sheet, the service temperature, and the sheet thickness, respectively.

**Figure 8:**
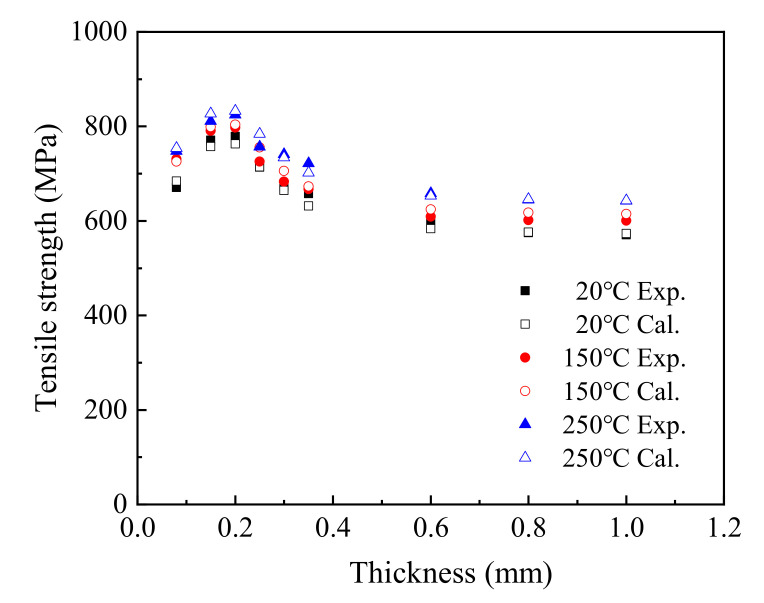
Comparison between experimental and calculated tensile strength.

The *T*–*t*–*Rm* equation was used to calculate the relationship among sheet thickness, service temperature, and tensile strength and compared with the experiment value, as shown in Figure 8.

In the range of 0.15–1.0 mm, the tensile stress calculated by the equation including temperature and thickness was consistent with the trend of the test curve, with values close to the test results. Comparing the experimental value and the fitted value, the maximum fitting deviation was 4.12% at 20 °C, 3.37% at 150 °C, and 3.57% at 250 °C. This indicates that it is reasonable to use *T*–*t*–*Rm* to predict the tensile strength of T2 copper sheet under different thicknesses and service temperatures.

#### 4.2.2. The Influence of Service Temperature on Yield Strength

**Figure 9:**
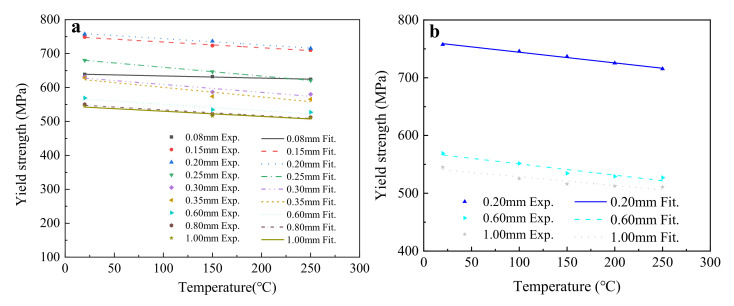
The influence of temperature on the yield strength of T2 copper sheet: (**a**) fitting results for three temperatures; (**b**) fitting results for five temperatures.

The average yield strengths of all groups of samples were fitted using the linear function given in Equation (6), and the fitting results are shown in Figure 9 and Table 7.

Figure 9 shows that the change laws of yield strength were the same for T2 copper sheets of different thickness, revealing a decreasing linear relationship with temperature. Table 7 indicates that the fitting results for parameters *a*_2_ and *b*_2_ using five temperatures were basically consistent with those using three temperatures, with a maximum fitting deviation of 4% for the 0.6 mm thick T2 copper sheet. Thus, the fitting results of the three temperatures were reliable and accurate. According to the fitting results of the slope *b*_2_, when the sheet thickness was less than 0.25 mm, the effect of temperature on the yield strength gradually increased with sheet thickness. On the contrary, when the sheet thickness was greater than 0.25 mm, it gradually decreased.

According to the *Rel*_0_ mentioned in Equation (3), the yield strength of the 1.0 mm thick T2 copper sheet could be obtained by substituting *y* = *Rel*_0_, *a*_1_ = 543.607, and *b*_1_ = 0.150 into Equation (6), as given in Equation (9).

(9) $$Rel_{t\;=\;1.0}=Rel_{0}=543.607-0.150T,$$

where *Rel*_0_ is the tensile strength of the T2 copper sheet, and *T* is the temperature.

Therefore, a service temperature–thickness–yield strength (*T*–*t*–*Rel*) empirical formula of T2 copper sheet could be established, as shown in Equation (10).

(10) $$Rel=543.607-0.150T+\left(\frac{7.083}{4{\left(t-0.178\right)}^{2}+0.032}\right),$$

where *Rel*, *T*, and *t* are the yield strength of the T2 copper sheet, the service temperature, and the sheet thickness, respectively.

**Figure 10:**
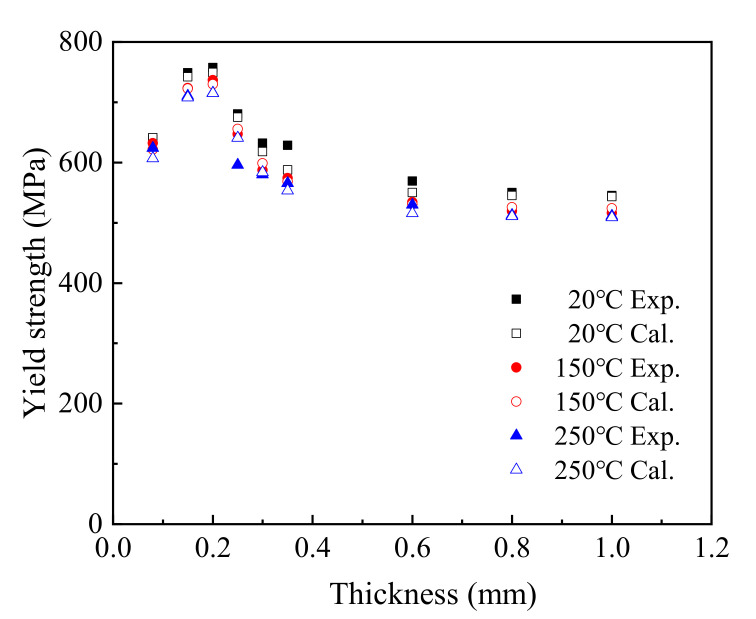
Comparison between experimental and calculated yield stress.

Figure 10 shows the yield strength obtained using the *T*–*t*–*Rel* formula compared with the experimental value.

When the thickness of the T2 copper sheet ranged from 0.08 mm to 1.0 mm, the yield strength calculated using the *T*–*t*–*Rel* equation approached the test values, with some values basically coinciding. Comparing the experimental values and fitted values, the maximum fitting deviation was 6.98% at 20 °C, 1.87% at 150 °C, and 7.56% at 250 °C. This shows that the *T*–*t*–*Rel* equation can be used to predict the yield strength of T2 copper sheet.

#### 4.2.3. The Influence of Service Temperature on Elongation

**Figure 11:**
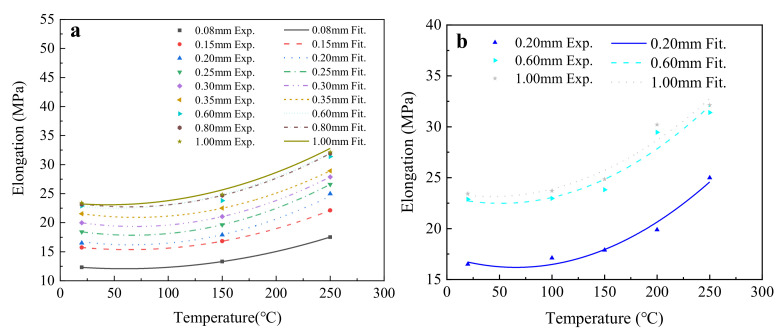
The influence of temperature on the elongation of T2 copper sheet: (**a**) fitting results for three temperatures; (**b**) fitting results for five temperatures.

According to the calculated results of the average elongation of each group of samples, the polynomial function given in Equation (11) was used to fit the experimental results, and the fitting results are shown in Figure 11 and Table 8.

(11) $$y=a_{3}\times T^{2}+b_{3}\times T+c,$$

where *y* is the elongation of T2 copper sheet, *a*_3_, *b*_3_, and *c* are the function parameters, and *T* is the temperature.

According to Figure 11 and Table 8, the elongation of T2 copper sheet changed in the same way for T2 copper sheets of different thickness. Generally, the elongation of T2 copper sheet increased as the temperature increased. Furthermore, the elongation increased with a smaller amplitude when the temperature was less than 150 °C, but increased with a larger amplitude when the temperature was larger than 150 °C.

Using the polynomial function, it was difficult to determine the reliability and reasonableness of the fitting results, because the *R^2^* of three temperatures had to be 1.0. Fortunately, the fitting results of five temperatures revealed that the elongation change of T2 copper sheet conformed to a polynomial function.

The elongation of the 1.0 mm thick T2 copper sheet could be obtained by substituting *y* = *δ*_0_, *a*_3_ = 2.231 × 10^−4^, *b*_3_ = −0.026, and *c* = 23.548 into Equation (11), as given in Equation (12).

(12) $$\delta_{t\;=\;1.0}=\delta_{0}=2.231\times 10^{-4}T^{2}-0.026T+23.548,$$

where *δ*_0_ is the elongation of the 1.0 mm thick T2 copper sheet and *T* is the temperature.

By combining Equations (5) and (12), a service temperature–thickness–elongation (*T–t–δ*) empirical formula of T2 copper sheet could be obtained, as shown in Equation (13).

(13) $$\delta =2.231\times 10^{-4}T^{2}-0.026T+23.548-19.585\times 0.005^{t},$$

where *δ* is the elongation, *T* is the service temperature, and *t* is the sheet thickness.

**Figure 12:**
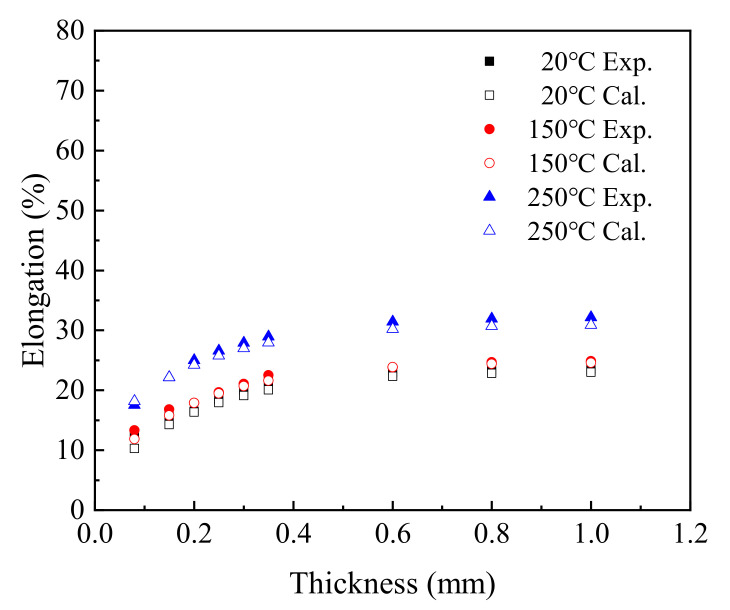
Comparison between experimental and calculated elongation.

Figure 12 is a comparison between the elongation obtained by experiment and the elongation calculated using the *T*–*t*–*δ* formula.

In the range 0.08 mm to 1.0 mm, the elongation calculated using the *T*–*t*–*δ* equation increased with the increase in thickness, and the trend was consistent with the test values under different service temperatures. Comparing the experimental values and fitted values, the maximum fitting deviation was 19.51% at 20 °C, 12.30% at 150 °C, and 3.94% at 250 °C. Therefore, the *T*–*t*–*δ* equation can be used to predict the elongation of T2 copper sheets under different temperature and thickness.

## 5. Conclusions

In the present work, T2 copper sheets of nine different thicknesses were tested at different temperatures. The experimental results were analyzed using different fitting methods to reveal the change laws of the tensile strength, yield strength, and elongation, and an empirical prediction model was established. The main conclusions are summarized as follows:

(1) The sheet thickness of T2 copper had an obvious influence on its tensile properties. Both the tensile and the yield strengths of the T2 copper first increased rapidly, then decreased sharply, and finally stabilized with the increase in sheet thickness. The elongation gradually increased and then stabilized with the increase in sheet thickness.

(2) Temperature had a significant effect on the tensile properties of materials, and the change laws of the tensile properties were almost the same for T2 copper sheets of different thickness. The tensile strength and elongation increased with temperature. On the contrary, the yield strength of T2 copper decreased linearly with temperature.

(3) Relationships between tensile strength and thickness and between yield strength and thickness conformed to the Lorentz function. However, there was an exponential relationship between elongation and thickness. Both tensile strength and yield strength were linearly related to temperature, while the relationship between elongation and temperature conformed to a polynomial function. Three empirical formulas referring to *T*–*t*–*Rm*, *T*–*t*–*Rel*, and *T*–*t*–*δ* were proposed and established on the basis of the abovementioned relationships. According to the fitted values close to the experimental values, *T*–*t*–*Rm*, *T*–*t*–*Rel*, and *T*–*t*–*δ* can be used as empirical prediction models.

## Figures and Tables

**Table 1:** Chemical composition of the tested thin T2 copper sheets (wt.%).

Cu	Bi	Sb	As	Fe	Pb	S	Others
99.95	0.0008	0.0002	0.0002	0.005	0.0003	0.005	≤0.1

**Table 2:** Number of specimens according to sample grouping.

Thickness	20 °C	100 °C	150 °C	200 °C	250 °C
0.08 mm	5	/	5	/	5
0.15 mm	5	/	5	/	5
0.20 mm	5	5	5	5	5
0.25 mm	5	/	5	/	5
0.30 mm	5	/	5	/	5
0.35 mm	5	/	5	/	5
0.60 mm	5	5	5	5	5
0.80 mm	5	/	5	/	5
1.0 mm	5	5	5	5	5

**Table 3:** Fitting results of tensile strength of T2 copper sheet under different temperature conditions.

Temperature	20 °C	150 °C	250 °C
*y*_0_ (MPa)	576.9	596.5	641.3
*A* (MPa·mm)	68.234	75.766	71.903
*t*_0_ (mm)	0.183	0.176	0.179
*ω* (mm)	0.211	0.235	0.248
*R* ^2^	0.978	0.993	0.976

**Table 4:** Fitting results of yield strength of T2 copper sheet under different temperature conditions.

Temperature	20 °C	150 °C	250 °C
*y*_0_ (MPa)	552.529	518.342	515.921
*A* (MPa·mm)	62.065	64.170	59.119
*t*_0_ (mm)	0.182	0.176	0.175
*ω* (mm)	0.186	0.180	0.175
*R* ^2^	0.977	0.993	0.970

**Table 5:** Fitting results of elongation of T2 copper sheet under different temperature conditions.

Temperature	20 °C	150 °C	250 °C
*y*_0_ (%)	23.694	25.004	32.179
*a*	18.263	19.398	21.085
*b*	0.006	0.006	0.004
*R* ^2^	0.986	0.993	0.999

**Table 6:** Fitting results of the relationship between temperature and tensile strength.

Thickness	*a*_1_ (MPa)	*b*_1_ (MPa·°C ^1^)	*R* ^2^
0.08 mm	662.868	0.333	0.997
0.15 mm	766.644	0.173	0.994
0.20 mm	772.934/769.342	0.196/0.199	0.967/0.915
0.25 mm	706.288	0.183	0.901
0.30 mm	655.179	0.302	0.839
0.35 mm	644.622	0.267	0.802
0.60mm	589.037/586.375	0.241/0.265	0.801/0.816
0.80 mm	565.441	0.297	0.957
1.0 mm	561.055/563.204	0.309/0.316	0.971/0.954

Note: For 0.20 mm, 0.60 mm, and 1.0 mm thick copper sheets, the first value was obtained from three test temperatures, while the second value was the fitting result of five test temperatures.

**Table 7:** Fitting results of the relationship between temperature and yield strength.

Sheet Thickness	*a*_2_ (MPa)	*b*_2_ (MPa·°C ^1^)	*R* ^2^
0.08 mm	640.440	−0.063	0.983
0.15 mm	751.085	−0.169	0.989
0.20 mm	761.909/762.673	−0.182/−0.185	0.992/0.992
0.25 mm	685.424	−0.258	1.0
0.30 mm	632.320	−0.233	0.898
0.35 mm	628.641	−0.281	0.899
0.60 mm	569.825/570.216	−0.188/−0.194	0.923/0.933
0.80 mm	550.897	−0.168	0.928
1.0 mm	545.389/543.607	−0.152/−0.150	0.908/0.898

Note: For 0.20 mm, 0.60 mm, and 1.0 mm thick copper sheets, the first value was obtained from three test temperatures, while the second value was the fitting result of five test temperatures.

**Table 8:** Fitting results of the relationship between temperature and elongation.

Thickness	*a*_3_ (×10^−4^)	*b*_3_	*c*	*R* ^2^
0.08 mm	1.497	−0.018	12.602	1.0
0.15 mm	1.946	−0.025	16.151	1.0
0.20 mm	2.616/2.475	−0.034/−0.033	17.062/17.265	1.0/0.975
0.25 mm	2.612	−0.035	19.023	1.0
0.30 mm	2.612	−0.036	20.607	1.0
0.35 mm	2.484	−0.035	22.147	1.0
0.60 mm	2.687/2.423	−0.034/−0.025	23.632/23.121	1.0/0.936
0.80 mm	2.656	−0.033	23.813	1.0
1.0 mm	2.695/2.231	−0.034/−0.026	24.024/23.548	1.0/0.945

Note: For 0.20 mm, 0.60 mm, and 1.0 mm thick copper sheets, the first value was obtained from three test temperatures, while the second value was the fitting result of five test temperatures.

## Data Availability

The data presented in this study are available on request from the corresponding author.
